# Love Wave Sensor with High Penetration Depth for Potential Application in Cell Monitoring

**DOI:** 10.3390/bios12020061

**Published:** 2022-01-24

**Authors:** Pedro A. Segura Chávez, Jérémy Bonhomme, Mohamed Lamine Fayçal Bellaredj, Lucile Olive, Denis Beyssen, Mourad Oudich, Paul G. Charette, Frédéric Sarry

**Affiliations:** 1 Laboratoire Nanotechnologies et Nanosystèmes (LN2—IRL 3463), Institut Interdisciplinaire d’Innovation Technologique (3IT), 3000 Boulevard de l’université, Sherbrooke, QC J1K OA5, Canada; bonhomme@insp.jussieu.fr (J.B.); paul.g.charette@usherbrooke.ca (P.G.C.); 2 Institut Jean Lamour, F-54000 Nancy, France; mohamed.bellaredj@univ-lorraine.fr (M.L.F.B.); lucile.olive@univ-amu.fr (L.O.); denis.beyssen@univ-lorraine.fr (D.B.); mourad.oudich@univ-lorraine.fr (M.O.); 3 Center for Acoustics and Vibration, The Pennsylvania State University, University Park, PA 16802, USA

**Keywords:** love wave, viscosity, L-SAW, mechanical properties, cell monolayers, transmission line model (TLM), surface acoustic waves (SAW), QCM

## Abstract

Love wave (L-SAW) sensors have been used to probe cell monolayers, but their application to detect changes beyond the focal adhesion points on cell monolayers, as viscosity changes on the cytoskeleton, has not been explored. In this work we present for the first time a Love wave sensor with tuned penetration depth and sensitivity to potentially detect mechanical changes beyond focal adhesion points of cell monolayers. We designed and fabricated a Love wave sensor operating at 30 MHz with sensitivity to detect viscous changes between 0.89 and 3.3 cP. The Love wave sensor was modeled using an acoustic transmission line model, whereas the response of interdigital transducers (IDTs) was modeled with the Campbell’s cross-field circuit model. Our design uses a substrate with a high electromechanical coupling coefficient (LiNbO_3_ 36Y-X), and an 8-µm polymeric guiding layer (SU-8). The design aims to overcome the high insertion losses of viscous liquid environments, and the loss of sensitivity due to the low frequency. The fabricated sensor was tested in a fluidic chamber glued directly to the SU-8 guiding layer. Our experiments with liquids of viscosity similar to those expected in cell monolayers showed a measurable sensor response. In addition, experimentation with SaOs-2 cells within a culture medium showed measurable responses. These results can be of interest for the development of novel cell-based biosensors, and novel characterization tools for cell monolayers.

## 1. Introduction

**Figure 1:**
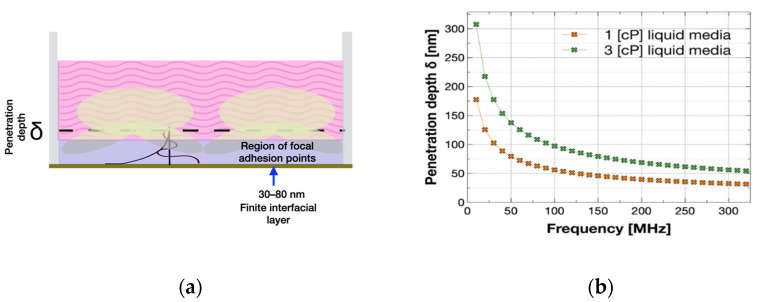
(**a**) Cell monolayer modeled by two-layer material that represents the focal adhesion region, and a bulk region beyond the focal adhesion: a finite interfacial layer (in violet) found between 30 and 80 nm, followed by a semi-infinite bulk region (in pink). This cell monolayer representation is based on the models used by H. Wu and Fang Li where cell viscosities are estimated in ranges between 1.5 and 2.2 cP [1,2,5]. (**b**) Penetration depth of horizontal polarized waves in liquid media as function of frequency for 1 cP and 3 cP viscosities in liquid media with a density of 1000 kg/m^3^. These graphs were computed with the formula $\delta =\sqrt{\frac{\eta_{liq}}{\rho_{liq}\pi f_{0}}}$ [4,6,7] where δ is the penetration depth, $\eta_{liq}$, the viscosity, $\rho_{liq}$ the density of the liquid, and $f_{0}$ the shear wave frequency. It can be seen that the penetration depth of waves in water-like liquid of 1 cP at 100 MHz is confined to around 50 nm, while for 30 MHz the penetration of acoustic waves is close to 100 nm.

Mechanical properties of mammalian cells have been studied and estimated (i.e., associated viscosity and Young modulus on simple viscoelastic layer models) with shear horizontal acoustic wave-based devices such as the quartz crystal microbalance (QCM) [1,2]. We hypothesize that the same experimental characterization can be performed using Love wave devices. Experiments with surface acoustic wave sensors and cell monolayers have shown that shear acoustic waves can reach the focal adhesion points of the cells that are the points of contact between the cells and the sensor surface (Figure 1a). Depending on the sensor’s frequency, the waves can also reach a deeper region beyond the focal adhesion points. The depth that waves can reach from the surface of the sensor is designated by the penetration depth [3,4], and represents the main zone of interaction of these acoustic sensors with a liquid or soft environment.

Figure 1a shows a simplistic schematic of a representation of a cell monolayer with an interfacial layer. The representation was used by Fang Li et al. [1] to consider a region of focal adhesion points and a region that represents the cell monolayer beyond the adhesion points. Fang Li et al. used this modeling approach of a cell monolayer in experiments with QCMs. Figure 1b shows the penetration depth as a function of frequency.

Important structures of cells such as the cytoskeleton in cell monolayers seeded on a surface can be found at a deeper distance from the surface than focal adhesion points. The cytoskeleton is one of the main structures governing the mechanical properties of mammalian cells. It was shown that the mechanical properties of cells are directly related to their condition, which enables the differentiation between types of cells [8]. In other studies, mechanical properties were used to differentiate between cancerous and normal cells [9,10], or to discern the age of cells [2]. The study of cells’ mechanical properties associated with the cytoskeleton structure is of great importance in cell mechanobiology [8]. This work motivates the demand for a Love wave sensor with high penetration depth for cell monolayer characterization.

**Figure 2:**
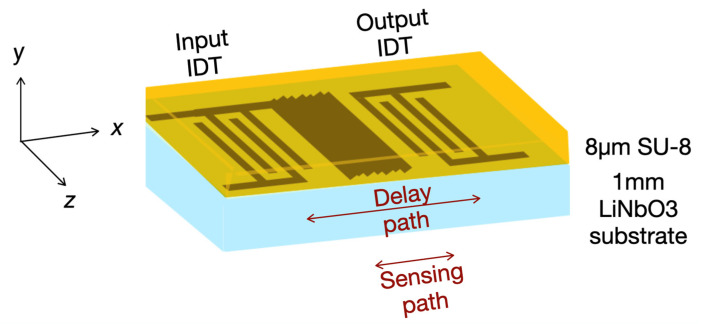
Schematic of a Love wave sensor. Love wave modes propagate from an input IDT to an output IDT. The L-SAW sensor has an 8-µm SU-8 guiding layer.

Love wave sensors use surface-wave-guided modes, known as Love modes, that propagate with shear-horizontal polarization (in-plane particle displacements) and are confined within a surface layer (Figure 2). Love wave sensors can be fabricated at low cost, and be easily patterned into arrays with microlithography techniques. Their working frequency is independent of the thickness of the piezoelectric substrate, and the acoustic waves can be generated by interdigital transducers. Thus, L-SAW sensors are seen to have great potential to reach the mass market in biosensor applications [11,12].

L-SAW sensors were used to investigate mammalian cell monolayers and detected focal adhesion of cells with typical frequencies between 80–300 MHz [3,11,13,14]. The use of sensors at these frequencies results in acoustic waves closely confined to the surface; i.e., the penetration depth cannot reach structures beyond focal adhesion points as shown by Saitakis et al. [3]. They showed that changes related to the cytoskeleton can be detected at a frequency of 35 MHz using QCMs but not with Love wave sensors of 110 MHz. Saitakis et al. also pointed out that the penetration depth should be the reason for the lack of response in their L-SAW sensor experiments.

In this work, we have designed and constructed a Love wave sensor with tuned penetration depth and sensitivity that aims to detect changes in cell monolayers beyond the penetration depth region, such as those reported with the QCM [3]. To this end, we propose a low frequency Love wave sensor of 30 MHz. Unlike a QCM, the frequency and sensitivity of a Love wave sensor does not depend on the thickness of its piezoelectric substrate. Our results may be of interest for the research and development of novel instrumentation to characterize cell monolayers as well as for the development of novel cell-based biosensors.

## 2. Materials and Methods

### 2.1. Love Wave Sensor Design Considerations

The low working frequency decreases the sensitivity of L-SAW sensors [15], thus a Love sensor requires a thicker guiding layer to work properly as a sensor due to the higher wavelength of the device. To overcome this issue, we designed a sensor with a polymeric guiding layer (8 µm SU-8), and a substrate with a high electromechanical coupling coefficient (LiNbO_3_ 36Y-X). 

We proposed the use of an 8-µm SU-8 guiding layer because it can be easily fabricated with conventional cleanroom equipment. The guiding layer we proposed had a dimensionless z value (guiding layer thickness over wavelength) of approximately 0.05, with high sensitivity. The sensitivity was expected because z = 0.05 lies in a highly sensitive region of the Love wave dispersion curve; we based our approach for the thickness optimization on the Love wave results of Roach et al. [16] and simulations with the transmission line model. In contrast, a typical SiO_2_ guiding layer would require a thicker guiding layer (z = 0.17, >>8 µm) [11] which would be technologically more complicated to deposit.

We chose LiNbO_3_ 36Y-X as the substrate material because its high electromechanical coupling coefficient allowed the immersion of IDTs covered by a polymer in liquids [17]. The substrate is efficient in its conversion from mechanical and electrical domains compared to other substrate options, such as quartz. On the other hand, the high electromechanical coupling coefficient allows the design of compact devices that require fewer finger electrodes in IDTs, which results in smaller delay paths (center-to-center distance of IDTs), which is desirable to further minimize the effects of the losses of a polymeric guiding layer. 

The Love wave sensor targets a viscosity range based on the cell monolayer viscosities estimated with the QCM by Fang Li and Wu. Their estimations of cell monolayers’ mechanical properties are shown in Table 1. We used liquids of viscosities from 1 cP up to 3.3 cP to test our sensor. In our approach, the Young modulus was disregarded for the design. We fabricated the sensor with common cleanroom techniques and tested it with liquids of calibrated viscosities.

### 2.2. Sensor Simulation

The Love wave sensor model used was a transmission line model (TLM) that allowed calculation of the expected losses and phase shifts in a delay line sensor configuration. The IDT’s response was calculated with the Campbell’s cross-field circuit model [18].

**Figure 3:**
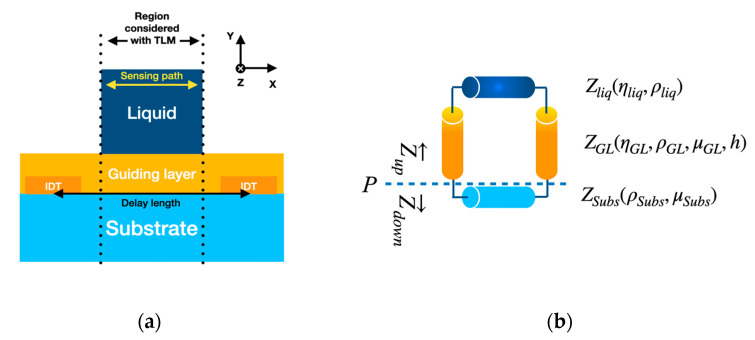
(**a**) Layered structure of a Love wave sensor made of three different materials: substrate rigid solid, a viscoelastic guiding layer, and a Newtonian liquid as the top layer. (**b**) A schematic of the transmission line circuit with the different impedances connected represents the Love wave sensor structure shown in (**a**); the physical parameters used for the characteristic impedance of each layer are also shown. Where *Z_liq_* is the impedance for a Newtonian liquid, *Z_GL_* the impedance for a viscoelastic guiding layer, and *Z_Subs_* the impedance for a rigid substrate, *η, ρ, μ* and *h* are, respectively, the viscosity, density, shear modulus, and thickness of the layer.

The acoustic transmission line model (TLM) considers the effects of viscosity in the layers that form the Love wave sensor (Figure 3a) [11,19]. The approach is mathematically analogous to the transmission lines of electromagnetic waves [20,21]. The TLM considers layers represented by a characteristic impedance given by the properties and type of the medium (Figure 3b). This medium can be viscoelastic solids, Newtonian liquids, or rigid solids. The propagation of the acoustic waves in the layers is described by Equations (1) and (2),

(1) $$\frac{d^{2}T_{J}}{dr^{2}}=Z\cdot Y\cdot v_{p},$$

(2) $$\frac{d^{2}v_{p}}{dr^{2}}=Z\cdot Y\cdot T_{J},$$

where r(x,y,z) represents the position of the particles [21] that compose the materials (*r*-axes are shown in Figure 2), $v_{P}\left(r\right)$ is the particle velocity, $T_{J}\left(r\right)$ is the shear stress in the J direction, *Z* is the impedance per unit length, and *Y* is the admittance per unit length. The layers considered are isotropic and homogeneous materials, and only in-plane displacements of the particles in the *z*-direction are considered.

The relationship between material properties (density (*ρ*), viscosity (*η*), and shear modulus (*μ*)) with the characteristic impedance (*Z_c_*), and the transmission line propagation factor (*k*) are shown in Equations (3)–(11) for the different types of materials.

Newtonian liquid

(3) $$Z_{c}=\sqrt{j\omega \rho \eta }$$

(4) $$k=-j\sqrt{\frac{j\omega \rho }{\eta }}$$

Rigid Solid

(5) $$Z_{c}=\sqrt{\rho \mu }$$

(6) $$k=\omega \sqrt{\frac{\rho }{\mu }}$$

Viscoelastic solid (Parallel elementary circuit model)

(7) $$Z_{c}=\sqrt{\frac{j\omega L}{G+j\omega C}}$$

(8) $$k=-j\sqrt{\omega L\left(G+j\omega C\right)}$$

(9) $$C=\frac{\mu }{\omega^{2}\eta^{2}+\mu^{2}}$$

(10) $$G=\frac{\eta \omega^{2}}{\omega^{2}\eta^{2}+\mu^{2}}$$

(11) L=ρ

The model considers the mechanical waves polarized in the z-direction (Figure 3a). In the sensor, the piezoelectric substrate is grounded in the surface between IDTs to reduce electrical effects, so they are omitted in the model [22].

The characteristic impedance of the transmission line was computed with the transmission line standard formula in Equation (12) [19,20]. *Z_load_* is the load impedance, *Z_char_* the characteristic impedance given by Equations (3)–(11) depending on the type and properties of the materials, and h is the thickness of the layer. The top layer and substrate are taken as perfectly matched impedances.

(12) $$Z=Z_{char}\frac{Z_{load}+Z_{char}\tanh\left(k_{y}h\right)}{Z_{char}+Z_{load}\tanh\left(k_{y}h\right)}$$

*k* is the wavenumber and *k_y_* (*k_y_* = *k*·cos(*ϕ*)) is the wavenumber value decomposed in the transverse resonance direction [11,19].

The transmission line model was numerically solved as an optimization problem that fulfilled the transverse (*Y* direction) resonance condition of the transmission line. For a complete description of the method, we refer to the review paper of Rocha Gaso et al. [11]. The sensor simulated with the transmission line model consisted of four material layers with properties shown in Table 2. The liquids’ properties, considered on the top of the sensor, are shown in Table 3.

### 2.3. Sensor Fabrication

We fabricated the L-SAW sensor in the cleanroom facilities. The sensor consisted of a lithium niobate (1 mm LiNbO_3_ 36Y-X, single-side SAW-grade polished) piezoelectric substrate, patterned metallic interdigital transducers (Au/Cr), a metallized sensing path (Au/Cr), and a guiding layer, as shown in Figure 2. The sensor was composed of an input IDT and an output IDT, respectively for the generation and detection of surface acoustic waves.

**Figure 4:**
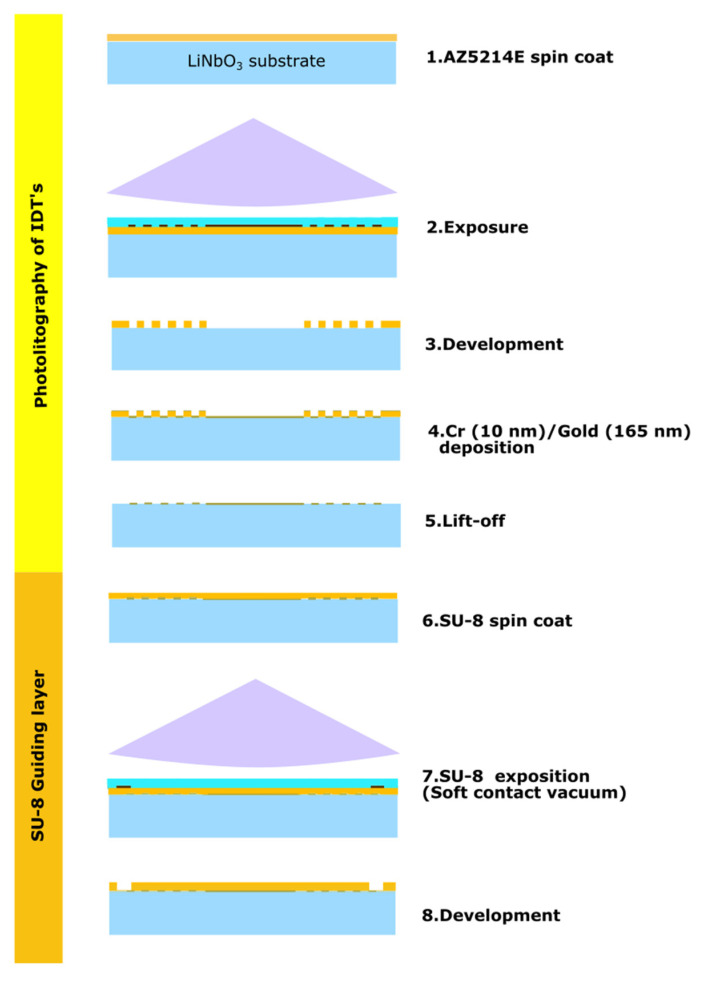
Microlithography fabrication process of the Love wave sensor. Steps for IDTs and metallization fabrication, followed by steps for SU-8 guiding layer coating and connection pad release. Parameters for the fabrication are shown in Table 4 and Table 5.

The transducers were made of simple IDTs orientated for propagation in the X-direction of the LiNbO_3_ 36Y substrate. The IDTs were composed of 12 fingers with a period of λ = 144 µm, 3 mm (21λ) aperture, delay path of 5.33 mm (37λ), and metallization-grounded sensing path of 2 mm (14λ). They were fabricated with microlithography patterning. The delay path was orientated to be coincident with the x-axis of the piezoelectric substrate. The sensor IDT design was implemented in the Layout Editor software 2019 (Juspertor GmbH, Unterhaching, Germany). Quartz-aluminum patterned lithography masks were used in this case. The main lithography process is shown in Figure 4.

A lift-off process was used for the development of the IDTs [25,26]. For the lithography we used the photoresist AZ5214E (MicroChemicals, Ulm, Germany), and mask aligner MJB4 (SÜSS MicroTec,, Garching, Germany). The IDT’s lithography steps are summarized in Table 4. After patterning the AZ5214E photoresist, thin film layers of chromium (10 nm) and gold (165 nm) were deposited on the piezoelectric substrate with the e-beam evaporator MEB 550 (Plassys Bestek, Paris, France) with a deposition rate of 0.15 nm/s. Sacrificial resist for lift-off was removed with rinsage on the universal photoresist stripper AZ-100 (Microchemicals, Ulm, Germany) for 45 min at 80 °C followed by a 5 min rinse with sonication, and a rinse in deionized water.

#### 2.3.1. SU-8 Guiding Layer Development

**Figure 5:**
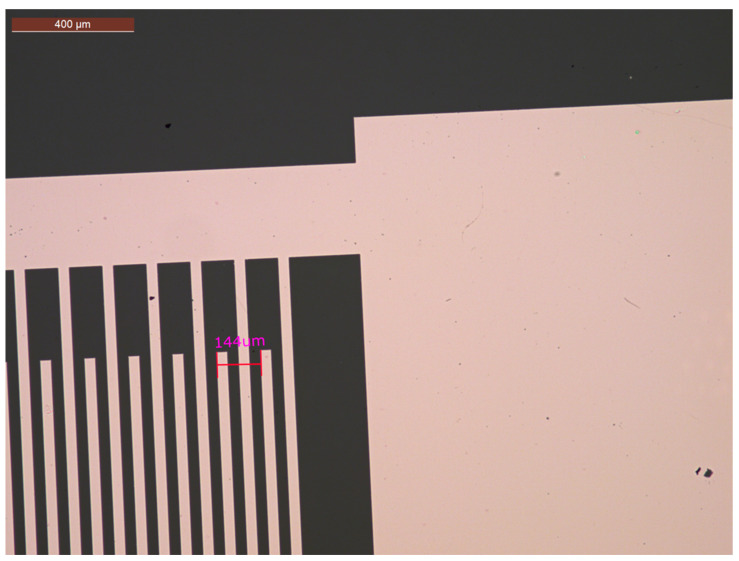
Micrograph of fabricated IDTs close to the metallization region between IDTs.

An 8-µm SU-8 guiding layer was deposited with spin coating and developed to release pads for electrical connection. SU-8 2015 (MicroChem, Westborough, MA, USA) was diluted with 8.8% *w/w* cyclopentanone. The spin coating followed the steps in Table 5. The development of the guiding layer required an additional mask with the guiding layer pattern, to release the pads for electrical connections. Figure 5 shows a device after the lithography and guiding layer (SU-8 transparent layer) process. 

#### 2.3.2. Fluidic Chamber

In order to hold liquids over the sensor, a custom fluidic chamber was developed, which consisted of a well made of PDMS glued directly to the SU-8 polymeric guiding layer, both materials being compatible with biosensor applications [16,27].

**Figure 6:**
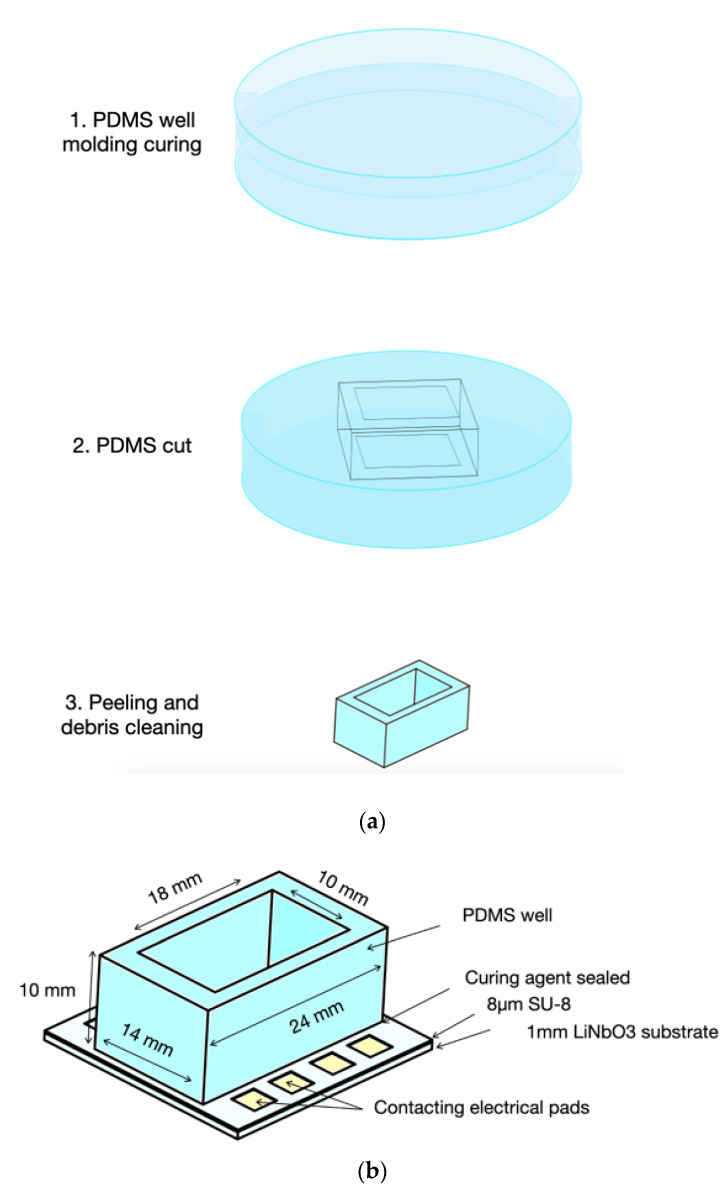
(**a**) Main fabrication steps for the PDMS fluidic chamber. (**b**) Schematic with dimensions of PDMS fluidic well glued to the Love wave sensor SU-8 guiding layer.

The fluidic chamber design is shown in Figure 6 and it supports volumes of at least 1 mL. The chamber volume was chosen to carry out experiments while diminishing high interference caused by the changing properties when evaporation occurs.

The fluidic chamber was prepared with PDMS (Sylgard™ 184 silicone elastomer kit Dow Corning, Midland, MI, USA) following the manufacturer’s recipe. The uncured polymer of the fluidic chamber was poured on a polystyrene petri dish of 55 mm in diameter and 10 mm in height. The poured polymer was degassed with vacuum and allowed to dry for three days at room temperature. Then, the fluidic chamber was carefully cut, according to the geometry shown in Figure 6, with a scalpel. Silicon debris was removed with air and deionized water (DIW), washed, and allowed to dry.

The PDMS well was glued directly to the surface of the SU-8 guiding layer. A thin layer of curing agent was gently spread on the PDMS base and gently pressed to the SU-8 surface to ensure contact between the surfaces. Then, the curing agent was allowed to dry for 48 h. The sensor was cleaned for 5 min with plasma oxygen at 100 W prior to experiments with living cells. 

### 2.4. Characterization Setup

**Figure 7:**
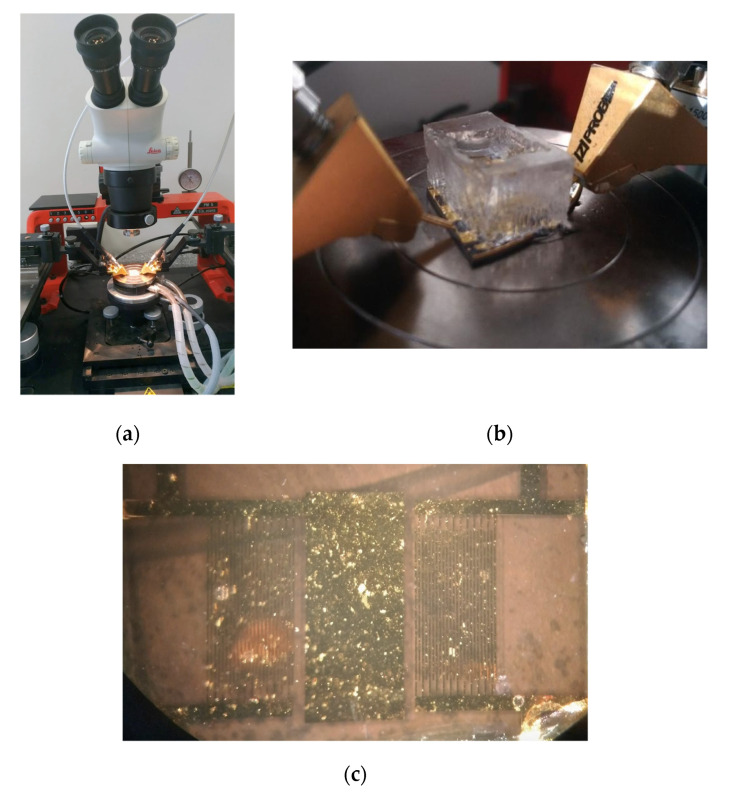
(**a**) The characterization system consisted of the probe station PM5 SÜSS MicroTec with holder temperature control, two rf-probes (Z-Probe P004 Cascade Microtech Inc., Cheshire, CT, USA), 50 ohm coaxial cables, and an Agilent-5230A vector network analyzer. (**b**) A fabricated sensor in the probe station during the characterization of glycerol-water solutions. The Z-probes are in contact with the IDT contact pads of the sensor. (**c**) A device fabricated during an experiment with cell culture media in the fluidic chamber.

We characterized the devices with RF probes connected to a vector network analyzer (Agilent-5230A, Santa Clara, CA, USA). A probe station with a controlled-temperature sample holder (PM5 SÜSS MicroTec, Garching, Germany), shown in Figure 7, was used to measure the scattering parameters (S_12_-parameters) on transmission of the Love wave sensor. The sensor S-parameters were measured and recorded for each 10 s during experiments. Each measurement consisted of a sample of 20,000 points of frequencies with a span from 10 MHz to 50 MHz. The s2p files were saved for each measurement and later were post-processed with the Python package scikit-rf [28,29]. The post-processing step helped us to filter noise, and discern both the phase at fixed frequency and the minimal insertion loss on the sensor. For noise removal, the S_12_ parameters were filtered by time-domain (gating function) [30]. The filtering kept the temporal ranges that corresponded to Love wave propagation from the input to the output port (the time window used considers this to be from 0.2 to 1.9 µs).

### 2.5. Glycerol Solutions Characterization

We tested the sensor’s sensitivity to liquids of adjusted viscosity with water-glycerol solutions. We prepared the viscous liquids based on the physical properties of water-glycerol mixtures to cover the target viscosity range [31,32]. The experiment temperature was fixed at 25 °C for the different mixtures and properties shown in Table 3. The fluidic chamber was filled with 1 mL of solution, which was carefully pipetted. To change between the different liquids in the chamber, the liquid in the chamber was removed and the chamber was then washed three times with deionized water.

### 2.6. Measurements with SaOs-2 Cells

Sensor measurements on cells were carried out using the SaOs-2 cell line. SaOs-2 cells (ATCC) were obtained from the Micro et Nanomécanique pour le Vivant group at l’Institut Jean Lamour. Cells were taken from subculture number 18, and incubated in culture flasks at 37 °C, in a 5% CO_2_, humidified atmosphere (MCO 18AC Incubator, Panasonic, Kadoma, Japan) for one day. The cell culture medium (CCM) was McCoy’s 5A modified liquid medium (ThermoFisher Scientific, Waltham, MA, USA) supplemented with 10% fetal bovine serum (FBS), 1% penicillin/streptomycin, 0.05% amphotericin B, and 1% L-glutamine.

Prior to each experiment, sensors were irradiated with UV light for 15 min on a cell culture safety hood. The experiments were carried out with the sensors connected to the characterization setup. The temperature of the probe station sample holder was set to 36.5 °C.

The experiments consisted of pipetting 1 mL of cell culture medium (without cells) into the fluidic chamber to acquire a reference level. Then, the CCM was exchanged for medium with cells. The CCM was removed with the pipette and 1 mL of cell culture medium with cells in a concentration of 150,000 cells/mL was pipetted. The responses of the sensors were followed during the experiment.

## 3. Results

### 3.1. IDT’s Response

**Figure 8:**
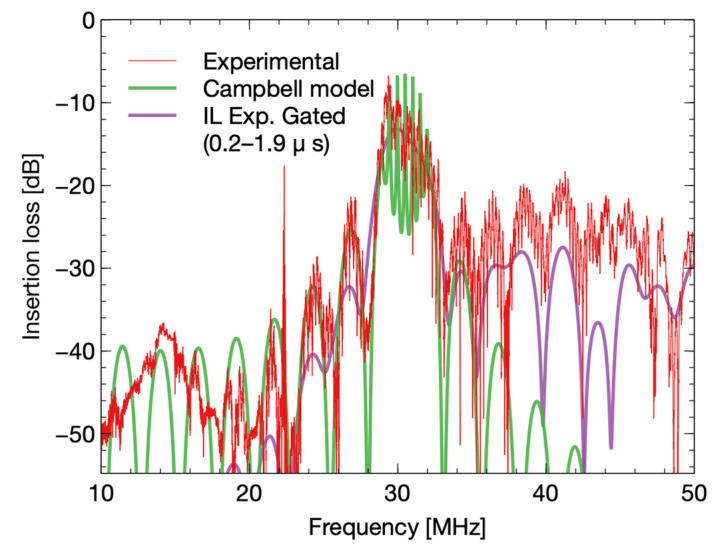
IDT response (green) of the Campbell model of a 30 MHz device on a LiNbO_3_ 36Y-X substrate, with symmetric 12 simple finger IDTs, and 29λ as a delay path. The red curve corresponds to the experimental response of the sensor on air. In purple is the experimental response with a gating filter, with a temporal window from 0.2 µs to 1.9 µs.

The IDT responses from simulation and the experiment are shown in Figure 8. The responses show noise variations at a frequency around 30 MHz due to triple transit effects of acoustic waves. Figure 8 shows the experimental response (purple curve) after filtering with a window of 0.2 µs to 1.9 µs (curve: IL Exp. Gated). The main noise variations were effectively removed with filtering in the time domain with an insertion loss level of −12 dB.

### 3.2. Model Response and Sensitivity of the Sensor

**Figure 9:**
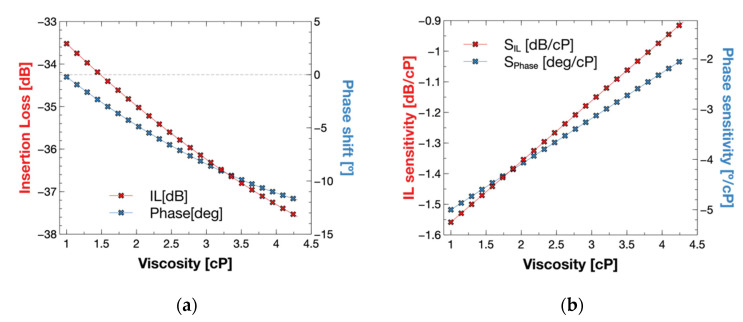
(**a**) TLM Insertion loss and phase model response of the sensor under changes in glycerol-water solutions from 1 cP to 4 cP. The model presents an increment of insertion loss and negative shift of the phase with solutions of higher viscosity. (**b**) Model sensitivity on the sensor for the different viscosities computed as the derivative of the responses.

The TLM simulation results are shown in Figure 9. We obtained highest and minimal insertion losses of −38 dB and −33 dB, respectively. It is seen that insertion losses became higher for liquids with high viscosity on top of the sensor. The phase angle at a frequency of 30 MHz shifted to the negative angles for higher viscosities. The sensitivity of the simulation takes values from −1.6 dB/cP to −0.9 dB/cP in insertion losses, and the phase angle takes values from −5°/cP to −2°/cP. 

The sensitivity value changed for the different viscosities as it decreased with high viscosities.

### 3.3. Response to Glycerol Solutions 

**Figure 10:**
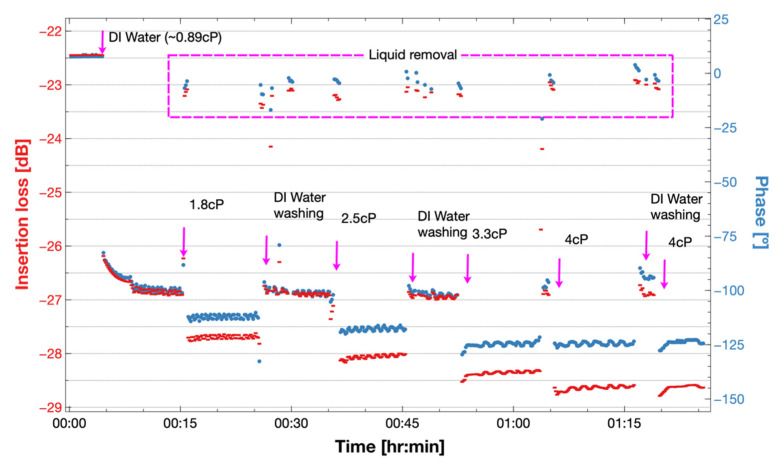
Experimental insertion loss and phase response of the Love wave sensor under changes of 1 mL glycerol-water solutions of 0.9, 1.8, 2.5, 3.3 and 4 cP at 25 °C on the PDMS fluidic well. We observed an increase of insertion loss and a negative shift in phase with solutions of higher viscosity and density.

The response of the sensor to viscous liquids is shown in Figure 10. It is observed that both phase and insertion loss shifted to negative values after insertion of a viscous solution, and that the values stabilized after a short time. At a time of around 5 min, the sensor loading was changed from air to a 0.89 cP liquid showing the highest shift in IL of −4.7 dB and in phase of −110°. After 10 min, the responses were stabilized at −26.8 dB and −100°. Around minute 15, the chamber liquid was removed, and the insertion loss level recovered to −23 dB and the phase to −5°. The response of the different viscosities shifted in the same direction but with higher values. Intermediate steps to wash the fluidic chamber with DIW are also shown in Figure 10. Between 0.89 cP and 4 cP, we depict a shift of 1.73 dB and 23° for the phase shift.

### 3.4. Measurements with Cells

**Figure 11:**
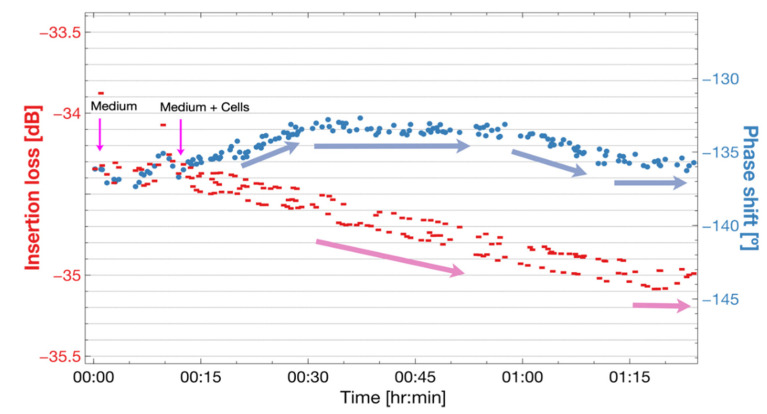
Experimental insertion loss and phase response of the Love wave sensor under changes of 1 mL of cell culture medium with SaOs−2 cells. Once the use of solution with cells commenced, an increase in the insertion losses was observed and an initial positive shift in the phase flipped to a negative shift.

The response of the sensor to cell culture medium with and without cells is shown in Figure 11. We observed a stable response with the sensor loaded with cell culture medium on both insertion loss and phase angle at the beginning of the experiment. The cell culture medium was changed for cell culture medium plus SaOs-2 cells after 10 min. The medium plus cells case showed that the insertion loss increased with time, while the phase shifted to positive phase angles. After 30 min the phase had stabilized at around −133°. After one hour of the experiment, the phase changed in the negative direction and the increasing rate of insertion losses was reduced.

## 4. Discussion and Conclusions

In the present work we designed and built a Love wave biosensor device for the detection of viscosity changes occurring in cell monolayers beyond the zone where focused adhesion with the sensor occurs. We showed for the first time that the Love wave sensor was sensitive to viscosity changes between 0.89 and 3.3 cP while presenting a high penetration depth (higher than ordinary Love wave biosensor depths) in viscous liquids due to the low operating frequency (30 MHz). Our results with viscous liquids tested viscosities of 0.89 cP (deionized water) and 4 cP, and showed a stable and repeatable response.

The experimental response of our sensor differed from previsions obtained with the transmission line acoustic model. The difference may have been caused by the fact that our model did not consider the electrical effects and that the grounding on the piezoelectric substrate surface between IDTs was not sufficient to avoid electrical interferences. Other possible reason could be a mismodeling of the viscoelastic nature of the guiding layer (viscoelastic model and/or values used in simulations). However, it was observed that the sensor response was similar to the results obtained from other Love sensors with substrates such as quartz (with a lower electromechanical coupling coefficient), where the direction of the shifts in both insertion loss and phase angle were in the negative direction [33,34] as the viscosity increased. Our model and experimental results were coincident in the direction of the shifts, and on the decrease in sensitivity at higher viscosity levels; the phase shifts from 3.3 cP to 4 cP were noticeably smaller with respect to the earlier viscosity shifts between 1.8 cP and 2.5 cP.

The results from the IDT model and the experimental response were in good agreement. The filtering removed significant noise and maintained both insertion loss levels and the shape of transfer function similar to the original response before filtering. In addition, we observed that the experiment’s responses for different viscous liquids stabilized well with the filtering technique.

Our experiment with cells demonstrates that the sensor and fluidic chamber enables experimentation with cells. We observed higher insertion losses with the cell medium. When SaOs−2 cells were deposited, a clear increase in the insertion loss was observed. 

After one hour, the response showed a decrease in the rate of increment of insertion losses. This insertion loss behavior is similar to what has been reported when seeding cells in high frequency Love wave sensors [3,13,35]. This response is due to the sensor surface becoming covered by the cells adhering to the sensor surface. However, the changes observed for the phase angle in the positive direction could be due to effects other than the adhesion, such as electrical interferences or mechanical interferences beyond the focal zone of adhesion; this needs to be clarified in future experiments.

The discrepancies of the sensor model with the experiment can be addressed using a fluidic cell design that avoids liquid over the IDTs, and/or implementing a complex model that considers electrical effects. For the experiment with cells, considering the complexity of the problem, we limited our result to prove that our sensor design kept measurable responses under cell sedimentation despite the low frequency used. We also acknowledge that the interpretation of acoustic shear sensors with cells is still a complex open problem under investigation [36]. To demonstrate the detection of mechanical changes in the cell cytoskeleton with the Love wave sensor, a proper characterization is needed, along with the construction of an adequate experimental design for that purpose.

Our work shows for the first time that Love wave sensors of high penetration depth can be sensitive enough to detect viscosity changes beyond the focal adhesion zone in cell monolayers, which is of potential interest for the development of cell-based biosensors, and of novel characterization tools for cell monolayers.

## Figures and Tables

**Table 1:** Estimated properties for cell monolayers with QCM by Fang Li and Wu.

Type of Experiment	Viscosity cP	Rigidity kPa	References
Confluent cell monolayer of MC-3T3 cells	1.52–1.56	48–49	[1]
Confluent cell monolayer of young TSC cells	1.86	0.9	[5]
Confluent cell monolayer of aged TSC cells	2.17	21	[5]

**Table 2:** Material properties for simulation of the Love wave sensor.

Layer	Material	Density (*ρ*) $\frac{kg}{m^{3}}$	Shear Modulus (*μ*) GPa	Viscosity (*η*) Pa·s	Layer Thickness (*h*) µm
Substrate	LiNbO_3_	4650	86.3	0	-
1	Cr	7190	115	0	0.010
2	Au	19,300	28.5	0	0.165
3	SU-8	1100	1.21	0.12 *	8

Properties taken from [21,23,24]. The SU-8 guiding layer is considered as a viscoelastic material represented by a parallel circuit [11]. * This was an approximation due to the lack of literature on the value of an SU-8 viscosity at 30 MHz.

**Table 3:** Glycerol-water solutions used at 25 °C.

Solution	Viscosity (*ρ*) cP	Density (*ρ*) $\frac{kg}{m^{3}}$	Glycerol *w/w* %
water	0.893	997	0
1.8 cP	1.8	1058.2	25.2
2.5 cP	2.5	1082	34.4
3.3 cP	3.3	1100	41.2
4 cP	4	1111.4	45.44

**Table 4:** Lithography recipe to process the IDTs on a 36Y cut LiNbO_3_ substrate.

Step	Value	Time
AZ5214E Spin coating	4000 rpm	40 s
Hotplate step	110 °C	66 s
Alignment and exposition	MJB4 Hg-Lamp 7 mW/cm²	4.7 s
Hotplate inversion step	120 °C	140 s
UV flood exposure	MJB4 Hg-Lamp 7 mW/cm²	35 s
Development	AZMIF726 Developer	20 s

**Table 5:** SU-8 guiding layer recipe.

Step	Value	Time
Plasma oxygen on substrate	100 W	60 s
Primer HDMS spin coat	4000 rpm	40 s
Hotplate	115 °C	60 s
SU-8 spin coat	500 rpm + 6000 rpm	10 s + 60 s
Hotplate	115 °C + 150 °C	60 s + 170 s
Alignment and exposition *	MJB4 Hg-Lamp 7 mW/cm²	24 s
Hotplate	115 °C	215 s
Development	SU-8 Developer	60 s + 10 s
Rinse	IPA	30 s
Dry	N2	20 s
Hotplate	150 °C	60 min

* The alignment and exposition steps needed soft-contact exposition due to stresses induced on the substrate by the SU-8 layer (we observed that the stress in a vacuum exposition tends to break the LiNbO_3_ wafers).
